# Cellular tropism and antigenicity of mink-derived SARS-CoV-2 variants

**DOI:** 10.1038/s41392-021-00617-0

**Published:** 2021-05-17

**Authors:** Li Zhang, Qianqian Li, Jianhui Nie, Ruxia Ding, Haixin Wang, Jiajing Wu, Xuguang Li, Xiaoming Yang, Weijin Huang, Youchun Wang

**Affiliations:** 1 Division of HIV/AIDS and Sex-transmitted Virus Vaccines, Institute for Biological Product Control, National Institutes for Food and Drug Control (NIFDC) and WHO Collaborating Center for Standardization and Evaluation of Biologicals, Beijing, China; 2 Centre for Biologics Evaluation, Biologic and Radiopharmaceutical Drugs Directorate, HPFB, Health Canada, Ottawa, ON Canada; 3 China National Biotec Group Company Limited, Beijing, China

**Keywords:** Vaccines, Infection

**Dear Editor**,

The outbreak of SARS-CoV-2 in minks has been observed recently, raising serious concerns over cross-species transmission and the emergence of variants capable of rendering antibody therapy and vaccines less effective. Here, the species tropism and antigenicity of the spike protein of ten variants were analyzed in pseudovirus-based assays involving 25 cell lines as well as 293T cells expressing ACE2 receptor from 14 species. No significant change in cellular tropisms was observed with the reported mink variants. There was a slight increase of infectivity in 69-70del and A262S-containing variants, and significantly reduced infectivity of the cluster 5 variant. In neutralizing assays, variants bearing Y453F, F486L, and A262S demonstrated decreased reactivities to at least one monoclonal antibody (mAb). Notably, variants with F486L and other additional mutations were resistant to eight neutralizing mAbs in addition to some polyclonal antisera or convalescent plasma. Together, these findings indicate that these variants are similar to the human viral isolates in terms of infectivity and cellular tropisms, while decreased sensitivity of variants bearing F486L in conjunction with other mutations to neutralization by some mAbs and polyclonal antibody preparations warrants close monitoring of the ever-evolving viruses.

SARS-CoV-2, the causative agent of COVID-19, is capable of infecting humans, along with a variety of other animal species. In April 2020, outbreaks of SARS-CoV-2 infections with fatal outcomes were first noted in minks (*Neovison vison*) in Noord-Brabant, Netherlands. The diseased animals demonstrated significant pathological changes such as acute severe interstitial pneumonia or diffuse alveolar damage,^1^ while deep sequencing analyses revealed that the mink infections were initially introduced from humans, with back transmissions to humans observed thereafter.^2^ To date, at least eight countries have witnessed SARS-CoV-2 outbreaks in mink farms. On Nov 5, the Danish public health authorities reported the detection of a mink-associated unique SARS-CoV-2 variant with four amino acid changes in the spike protein (referred to as “Cluster 5”) in 12 human cases.^3^ Importantly, the decreased reactivities of the variants to convalescent plasma is worth noting, given the potential implication on cross-species transmission, antibody therapies, and vaccine efficacy.^3^

In this study, we analyzed 338 mink-derived SARS-CoV-2 sequences reported to GISAID (up to December 2, 2020); they include 13 sequences from *Mustela lutreola* and 325 from Neovison Vison (Supplementary Table 1). The variants were compared with the currently dominant D614G variant, which were found to be more infectious than the early isolates^4,5^ and present in most of the variants in this study. Nine mutations, 69-70del, G261D, A262S, Q314K, L452M, Y453F, F486L, I692V, M1229l, along with D614G, were found in the Spike protein of the mink variants (Supplementary Table 1). These variants bear either Y453F or F486L, along with other mutations (Supplementary Table 2). All of the variants mentioned in the manuscript were default to contain D614G mutation, except some variants marked as G614D. The order of prevalence in mink is as follows: F486L+A262S+Q314K (28%), Y453F+69-70del (21%) and D614G (21%), F486L+L452M (10%), Y453F+G614D (8%), Y453F (2%), Y453F+G261D+G614D (2%), F486L (2%), and Y453F+69-70del+I692V+M1229I (2%). With respect to the prevalence of Y453F and F486L variants in the human population, we found 22 human isolates with F486L mutation (mainly from the Netherlands) and 328 isolates with Y453F mutations (mostly from Denmark). Based on the sequence information, we constructed 10 pseudoviruses carrying these natural mutations (Supplementary Table 1). They are Y453F, Y453F+69-70del, Y453F+69-70del+I692V+M1229I (the cluster 5 variant), Y453F+69-70del+S1147L, Y453F+G614D, Y453F+G261D+G614D, F486L, F486L+L452M, F486L+A262S+Q314K, and A262S.

We next analyzed the infectivity of these pseudoviruses. To this end, we employed cell lines derived from a wide range of species, as this would allow us to gain insight into both the infectivity and tropisms of these natural variants. Specifically, 25 cell lines from 10 different species and 293T cells expressing angiotensin-converting enzyme 2(ACE2) from 14 different species were used in the well-established assay reported by us recently.^4^ Positive reaction was set as readings over 10^4^ RLU (relative luminescence unit) by subtracting the background (Supplementary Fig. 1). We found 8 out of 25 cell lines from human or primates were susceptible to the infection by these variants; it is of note that slightly increased infectivity of 69-70del and A262S bearing variants was observed whereas significantly reduced infectivity was associated with the cluster 5 variant (Fig. 1a, b and Supplementary Figs. 1 and 2). Variants carrying single Y453F or F486L mutation have similar infectivity compared with D614G.

**Fig. 1:**
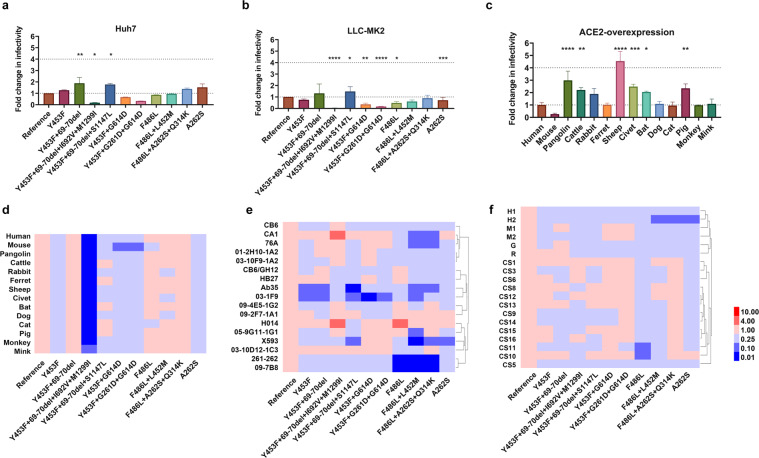
The cells were infected with an equal amount of 11 pseudoviruses. The luminescence activity was analyzed 24 h after the infection. The mean ratio of the RLU values of mink-derived variant to the reference strain (D614G) was calculated and presented in **a**–**d**. **a** Infection of human cell line by the mink variants. **b** Infection of monkey cell line by the mink variants. **c** Infection of reference strain (D614G) in different ACE2 over-expressed 293T cells. **d** Infection of reference and ten variants in 14 different ACE2 over-expressed 293T cells (heatmaps). **e** Antigenicity analysis of the mink mutants in 17 monoclonal antibodies (heatmaps). **f** Antigenicity analyses of mink variants with polyclonal antibodies (heatmaps). CS denotes: convalescent plasma, H: horse, G: goat M: mouse. Data represent the ratio of variants/D614G in EC_50_ (**e**, **f**). The red and blue boxes indicate the increase or decrease of the infectivity (**d**) or neutralization activity (**e**, **f**) as shown in the scale bar. Three to six times experiment results were included

As most of the cell lines were not susceptible to infections by D614G or variants, 293T cells with over-expressed 14 different species of ACE2 were used for further analyses of infectivity. As shown in Fig. 1c. The D614G reference strain failed to infect 293T cells expressing mouse ACE2, and demonstrated similar infectivity to cells expressing ACE2 from mink, ferret, dog, monkey, cat, or human (Fig. 1c). However, increased infectivity was even more significant in cells expressing pangolin, cattle, rabbit, sheep, civet, bat, and pig ACE2 compared to that of human ACE2 (Fig. 1c). Moreover, Y453F+69-70del variant was slightly more infectious (<4-times) than D614G, in contrast, cluster 5 was significantly less infectious (Fig. 1d and Supplementary Fig. 3). Given no significantly increased infectivity was found with these variants, it is unlikely the tropisms were altered. Nonetheless, mink cell line MV1-Lu, with overexpressing of mink ACE2, could not be infected with the viruses (Supplementary Fig. 4), suggesting other host factors essential SARS-CoV-2 infection could be absent in MV1-Lu.

The antigenicity of these variants was subsequently investigated. We first tested the 17 monoclonal antibodies (mAbs) targeting receptor-binding domain (RBD) of the spike protein against the mink variants, and found various degrees of altered neutralizing activities compared to the reference D614G. As shown in Fig. 1e and Supplementary Fig. 5, mAbs Ab35 and 03-1F9 displayed significantly reduced neutralizing activity against the mink Y453F variants, while mAb X593 completely lost its neutralizing activity against A262S. Furthermore, 8 out of 17 mAbs have reduced neutralizing activity against variants carrying single F486L or additional mutations, with abolished neutralizing activities found in mAb 261-262 and 09-7B8 (Fig. 1e and Supplementary Fig. 5). Finally, we determined the susceptibility of these variants to neutralization by several sources of polyclonal antibodies. These antibody preparations include sera from animals immunized with RBD (horses and rabbits), full-length S protein and inactive virus (goat), and DNA-expressing full-length S gene (mice), and convalescent serum from 13 patients with COVID-19. As shown in Fig. 1f and Supplementary Fig. 6, variants with single Y453F and additional mutations including the 69-70 deletion demonstrated no altered susceptibility to polyclonal antibodies derived from animals immunized with the spike immunogens and convalescent plasma from COVID-19 patients, while various degrees of resistance to polyclonal antibodies was observed with variants bearing single F486L or additional mutations, specifically, one of two equine sera and 2 of 13 human convalescent sera (Fig. 1f and Supplementary Fig. 6).

In short, we found no evidence that mink variants have altered tropisms compared to the currently predominant D614G variant. Moreover, 69-70 del variants did not appear to become resistant to RBD-specific mAb and polyclonal antibodies from animals and convalescent patients. It is of note that the recently surging variants of B.1.1.7 lineage in the UK carry multiple mutations including the 69-70 del studied here. More studies would be needed to better understand the impact of 69-70 del mutation on the transmission and antigenicity of the B.1.1.7 variants. Furthermore, the decreased sensitivity of the F486L variants to some neutralizing mAb and selected convalescent plasma warrants further investigations, given the implication on the formulation of mAb-based antiviral therapies.

## Supplementary information

Supplementary Materials

The cell tropism of mink variants (25 cell lines)

Infection of 6 cell lines with mink variants

Infection of 293T cells expressing with ACE2 from different species

Infection of MV1-lu cells expressing with human, mink or ferret ACE2

The antigenicity analyses of mink variants using monoclonal antibodies

The antigenicity of mink related SARS-CoV-2 using polyclonal antibodies and convalescence plasma
