# Supplementary material for: Cellular tropism and antigenicity of mink-derived SARS-CoV-2 variants
Source: Signal Transduct Target Ther. 2021 May 17;6:196. doi: 10.1038/s41392-021-00617-0 (PMC8127491; doi:10.1038/s41392-021-00617-0)
Supplement: Supplementary file 1 — Supplementary Materials [file 41392_2021_617_MOESM1_ESM.docx]

Supplementary Materials for

**Cellular Tropism and Antigenicity of Mink derived SARS-CoV-2 variants**

Li Zhang, Qianqian Li, Jianhui Nie, Ruxia Ding, Haixin Wang, Jiajing Wu, Xuguang Li, Xiaoming Yang, Weijin Huang, and Youchun Wang

Correspondence to: [wangyc@nifdc.org.cn](mailto:wangyc@nifdc.org.cn), [huangweijin@nifdc.org.cn](mailto:huangweijin@nifdc.org.cn), or [yangxiaoming@sinopharm.com](mailto:yangxiaoming@sinopharm.com)

**This file includes:**

Materials and Methods

Supplementary Tables 1

Supplementary Figure legend. S1 to S6

**Materials and Methods**

**Sequence analysis**

As of December 2, 426 mink related SARS-CoV-2 isolates were downloaded from GISAID website. The ambiguous and redundant were removed. There were 338 complete S protein sequences, including 13 Mustala lutreola isolates and 325 Neovison vison isolates. The variants bearing Y453F and F486L, along with additional mutations with humans as host origin were also analyzed. There are 22 human isolates containing F486L mutation with complete S protein sequence; 328 of 342 human isolates containing Y453F mutation with complete S protein. The reference sequence used in this paper is D614G variant which constitutes over 85% of the total sequence in the GISAID database.

**Plasmid**

The SARS-CoV-2 spike (GenBank: MN908947) protein expression plasmid pcDNA3.1-SARS-CoV-2-Spike was optimized by mammalian codon optimization, and constructed on the eukaryotic expression vector pcDNA3.1 between BamHI and XhoI. The point mutation plasmid was constructed based on pcDNA3.1-SARS-CoV-2-Spike using the method described in our previous paper4. The specific mutation sites and corresponding primers are presented in the table below.

Fourteen ACE2 protein expression plasmids were constructed, including Human (BAB40370.1), Mink (QNC68911.1), Dog (MT663955), Cat (MT663959), Pangolin (XP_017505746.1), Pig (NP_001116542.1), Mouse (ABN80106.1), Bat (KC881004.1), Cattle (NP_001019673.2), Rabbit (MT663961), Ferret (MT663957), Sheep (XP_011961657.1), Civet (AY881174.1) and Money (MT663960). All the sequence was optimized by mammalian codon, and flag tag (dykddddk) was added to the C-terminal. The nuclear acid was synthesized in general biosystems Co (Beijing China), and constructed into the eukaryotic expression vector pcDNA3.1 between BamHI and XhoI.

| Name | Oligonucleotides | |
| --- | --- | --- |
| 69-70del-F | | GTGACCTGGTTCCACGCCATCAGCGGCACCAATGGCACCAAGAG |
| G261D-F | CGTCAAGCGGTTGGACCGCTGATGCTGCGGCATATTACGTG | |
| A262S-F | GGACCGCTGGAAGCGCGGCATATTACGTGGGCT | |
| Q314K-F | GTGGAGAAGGGCATCTACAAGACCAGCAATTTCAGAGT | |
| L452M-F | TTGGAGGCAATTACAATTACATGTACAGACTGTTCAGAA | |
| Y453F-F | GTTGGAGGCAATTACAATTACCTGTTCAGACTGTTCAGAAAGAGCAATC | |
| F486L-F | GTAATGGCGTGGAGGGCCTGAATTGCTACTTCCCTCTGC | |
| D614G-F | GTGGCCGTGCTGTACCAGGGCGTGAATTGCACCGAGGT | |
| I692V-F | CAGAAGCGTGGCCAGCCAGAGCGTGATCGCCTACACCATGAGCCTG | |
| S1147L-F | TGCAGCCTGAGCTGGACCTGTTCAAGGAGGAGCTGGAC | |
| M1229I-F | CATCGCCGGCCTGATCGCCATCGTGATCGTGACCATCATGCTGTGCTGCA | |

**Cells**

293T-hACE2 (constructed by our laboratory), 293T (American Type Culture Collection [ATCC], CRL-3216), Huh-7 (Japanese Collection of Research Bioresources [JCRB], 0403), HepG2 (ATCC, HB-8065), HeLa (ATCC, CCL-2), MRC-5 (ATCC, CCL-171), A549 (ATCC, CCL-185), Hep2(ATCC, CCL-23), Vero (ATCC, CCL-81), Vero E6 (ATCC, CRL-1586), LLC-MK2 (ATCC, CCL-7), ST(ATCC, CRL-1746), BHK21(ATCC, CCL-10), CHO (ATCC, CCL-61), NIH/3T3(ATCC, CRL-1658), RAW264.7(ATCC, TIB-71), MDCK (ATCC, CCL-34), Cf2TH(ATCC, CRL-1430), CRFK(ATCC, CCL-94), MDBK(ATCC, CCL-22) and MV1-Lu (ATCC, CCL-64) cells were cultured in Dulbecco’s modified Eagle medium (DMEM, high glucose; Hyclone). DC2.4 (Millipore, SCC142), K562 (ATCC, CCL-243) and MdKi (Laboratory of Dr. Zhengli Shi) cells were incubated in RPMI medium modified (Hyclone). RlKiT (Laboratory of Dr. Zhengli Shi) were cultured in DMEM/F-12, GlutaMAX (GIBCO). All the cells cultured media was supplemented with 100 U/mL of Penicillin-Streptomycin solution (GIBCO), 20mM N-2-hydroxyethylpiperazine-N-2-ethane sulfonic acid (HEPES, GIBCO) and 10% fetal bovine serum (FBS, Pansera ES, PAN-Biotech). Cells were cultured in a 5% CO_2_ environment at 37 °C and passaged every 2–3 days using 0.25% Trypsin-EDTA (GIBCO). The ACE2 transient overexpression cells were prepared by transfecting 293T cells with the same amount of plasmids of different species of ACE2 individually using Lipofectamine 2000 (Invitrogen) transfection reagent. After transfection, the cells were cultured in 5% CO_2_ environment at 37℃ for 24h, and used for the follow-up test.

**Monoclonal antibody**

There are 17 neutralizing monoclonal antibodies against SARS-CoV-2 spike protein used in this study. The CB6, CB6/GH12 and CA1 were provided by Dr. Jinghua Yan, H014, HB27 were provided by Dr. Liangzhi Xie, 76A, 261-262, and Ab35 were provided by Dr. Linqi Zhang, X593 was provided by X. sunney Xie, 03-1F9, 09-7B8, 09-4E5-1G2, 09-2F7-1A1, 01-2H10-1A2, 03-10D12-1C3, 03-10F9-1A2 and 05-9G11-1G1 were provided Beijing Biocytogen Co.

**Convalescent serum**

Thirteen serum samples of convalescent patients were collected from Wuhan (CS1, CS3, CS5, CS6, CS8, CS9, CS10) and Hunan Province (CS11, CS12, CS13, CS14, CS15 and CS16), respectively, provided by China National Biotech Group and Nanhua University. The study was performed in accordance with the Good Clinical

Practice and the Declaration of Helsinki principles for ethical research. Consent form was signed by each participant before serum collection.

**Polyclonal antibody and animal immunization**

Animal experiments were performed in accordance with institutional (NIFDC, Beijing, China) guidelines for laboratory animal care and use. The Animal Care and Use Committee at the NIFDC approved the study protocol (NIFDC-2020(B)-001).

Horses were immunized thrice with SARS-Cov-2 RBD protein every 10 days (3mg, 6mg, 12mg with an equal volume of Freund’s complete adjuvant). Sera were collected 7 days after third immunization (H1 and H2).

Ten SPF BALB/c mice were immunized with pcDNA3.1- SARS-Cov-2-Spike plasmid. Each animal was immunized with 50μg every two weeks for 3 times. Blood samples were collected 7 days after the third immunization. The serum samples from 5 mice were pooled and labeled as M1 and M2 respectively.

Goats were first immunized with 200μg recombinant full-length SARS-Cov-2-Spike protein expressed by insect cell system mixed with Freund's incomplete adjuvant. Two weeks later, 100 μg of purified SARS-CoV-2 inactivated virus (Vero cell culture) mixed with Freund's incomplete adjuvant were immunized again, the same dose of SARS-CoV-2 inactivated virus was immunized a week later. Serum was collected one week after the third immunization.

The SPF New Zealand white rabbits were immunized with 100 μg recombinant SARS-CoV-2 spike RBD protein mixed with Freund's complete adjuvant. The rabbits were re-immunized with 50 μg RBD protein and Freund's incomplete adjuvant on day 14 and 21. The sera were collected 7 days after the final immunization.

**Pseudovirus Preparation**

The pseudovirus of reference SARS-CoV-2 and mink derived variants were constructed using the method reported in our previous report^4^. Briefly, 293T cells were seeded into T75 cell culture bottles and incubate overnight in 5% CO_2_ incubator at 37℃. When the confluence reached 70-90%, cells were infected with 7.0 × 10^4^ TCID_50_ / ml G*ΔG-VSV (kerafast). 30μg S protein expression plasmid was transfected according to the user's manual. After 6-8 hours, the cells were washed twice with PBS + 1% fetal bovine serum, and cultured in fresh complete DMEM at 37℃ in 5% CO_2_ incubator. 24 hours later, the SARS-CoV-2 pseudovirus containing culture supernatant was harvested, filtered, aliquoted and frozen at - 70℃.

**Pseudovirus infection**

The RNA of SARS-CoV-2 pseudoviruses were extracted and reverse transcribed by RT-PCR. The copies of the pseudovirus were calculated as described previously4. Pseudovirus was then diluted to the same particle number and added into 96-well plate. After trypsin digestion, 2 × 10^4^ /100μl cells were added into each well. Cells were then incubation for 24 hours in a 37℃ incubator with 5% CO_2_. The chemiluminescence detection was performed using luciferase substrate (PerkinElmer). Each group contained 3 to 6 replicates.

**Neutralization test**

The inhibition effect of monoclonal antibodies polyclonal antibodies and convalescent sera on SARS-CoV-2 was evaluated by quantitative detection of the luciferase activity. Specifically, the test samples were serially diluted by 3-fold for at least 8 steps in the 96-well plate. The SARS-CoV-2 pseudovirus was then mixed with equal amounts of variants in separate wells, with controls including virus or cells only in each plate. The 96 well plates were then incubated at 37℃ for 1 hour. Afterwards, Huh7 cells were added to each well at the concentration of 2 × 10^4^ / 100μl/well. Following incubation at 37℃ for 24 hours in an atmosphere of 5% CO_2_. Luciferase activities were measure as described above, with EC_50_ being calculated using Reed Muench method.

**Statistical analysis**

All the experiments were repeated at least three times. Data were analyzed with graph prizm 8, and present as Mean ± SEM. One-way ANOVA and Holm-Sidak’s multiple comparisons test was used to do statistical analysis. * indicates P<0.05, ** indicates P<0.01, *** indicates P<0.005, and **** indicates P<0.001

**Supplementary Table 1**

**A. The mutations in mink epidemic SARS-CoV-2**

| **aa69** | | **aa70** | | **aa261** | | **aa262** | | **aa314** | | **aa452** | | **aa453** | | **aa486** | | **aa614** | | **aa692** | | **aa1229** | |
| --- | --- | --- | --- | --- | --- | --- | --- | --- | --- | --- | --- | --- | --- | --- | --- | --- | --- | --- | --- | --- | --- |
| **H** | **263** | **V** | **263** | **G** | **331** | **A** | **245** | **Q** | **254** | **L** | **306** | **Y** | **217** | **F** | **212** | **G** | **322** | **I** | **333** | **M** | **333** |
| **Del** | **75** | **Del** | **75** | **D** | **7** | **S** | **93** | **K** | **84** | **M** | **32** | **F** | **121** | **L** | **126** | **D** | **7** | **V** | **5** | **I** | **5** |

**B. The Y453F and F486L related mink and human SARS-CoV-2 variants**

|  |  | Mink | | | | Homo sapiens | |
| --- | --- | --- | --- | --- | --- | --- | --- |
|  |  | M. lutreola | | N. vison | | Human | |
|  |  | Netherlands | Denmark | Netherlands | Denmark | Netherlands | Denmark |
| 1 | Reference（614G） | 5 | 0 | 52 | 4 | / | / |
| 2 | Y453F | 0 | 0 | 6 | 1 | 0 | 78 |
| 3 | Y453F+69-70del | 0 | 0 | 0 | 60 | 0 | 162 |
| 4 | Y453F+69-70del+I692V+M1229I | 0 | 0 | 0 | 5 | 0 | 11 |
| 5 | Y453F+69-70del+S1147L | 0 | 0 | 0 | 0 | 0 | 35 |
| 6 | Y453F+G614D | 1 | 0 | 23 | 0 | 3 | 0 |
| 7 | Y453F+G261D+G614D | 4 | 0 | 3 | 0 | 0 | 0 |
| 8 | F486L | 0 | 0 | 7 | 0 | 1 | 0 |
| 9 | F486L+L452M | 0 | 0 | 29 | 0 | 5 | 0 |
| 10 | F486L+A262S++Q314K | 0 | 0 | 82 | 0 | 13 | 0 |
| 11 | A262S | 0 | 0 | 7 | 0 | / | / |

**Supplementary Fig 1**

The cell tropism of mink variants (25 cell lines). 25 cell lines were studied for their susceptibility to infection of the pseudoviruses. The amounts of virus were determined based on copy numbers of nucleic acid. The cells were harvested 24 hours after the infection. The mean value of RLU of three to six replicates were calculated and presented in the heatmap using Hem I. RLU vales <10^4^ was considered to be negative. The red and blue indicates the intensity of the signal (from the low to high) as shown in the scale bar. 3~7 denotes 10^3^~10^7^

**Supplementary Fig 2**

Infection of 6 cell lines with mink variants. RLU vales > 10^4^ were further analyzed for the relative infectivity compared to the reference virus in theses cell lines. The horizontal dashed lines indicate the threshold of 4-fold difference.

**Supplementary Fig 3**

Infection of 293T cells expressing with ACE2 from different species. Equal amount of different ACE2 plasmids were transfected to 293T cells and ACE2 expression was confirmed by FACS analyses by detecting the flag tag present in the C terminal of ACE2. Cells were than harvest to perform the infection study as described in the methods section, with the relative infectivity of mink variants compared to reference strains being presented.

**Supplementary Fig 4**

Infection of MV1-lu cells expressing with human, mink or ferret ACE2. Equal amount of different ACE2 plasmids were transfected to 293T cells. Cells were than harvest to perform the infection study as described in the methods section, with the RLU of mink variants was presented.

**Supplementary Fig 5**

The antigenicity analyses of mink variants using monoclonal antibodies.

mAbs were diluted serially and pre-incubated with the SARS-CoV-2 pseudoviruses at 37°C for one hour before added in to Huh-7 cell cultures. The ratio of EC_50_ between the variants and reference D614G was presented. The horizontal dashed lines indicate the threshold of 4-fold difference. The experiments were repeated at least 3 times.

**Supplementary Fig6**

The antigenicity of mink related SARS-CoV-2 using polyclonal antibodies and convalescence plasma. Serially-diluted antibody preparations were incubated with the variants. The rest of the procedure is the same as above.
