# Supplementary figures and images for: Cellular tropism and antigenicity of mink-derived SARS-CoV-2 variants
Source: Signal Transduct Target Ther. 2021 May 17;6:196. doi: 10.1038/s41392-021-00617-0 (PMC8127491; doi:10.1038/s41392-021-00617-0)

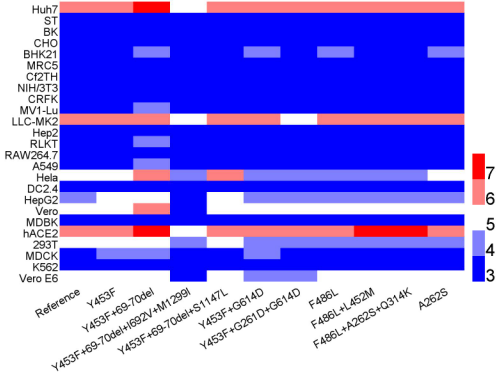

Supplement: Supplementary file 2 — The cell tropism of mink variants (25 cell lines) [file 41392_2021_617_MOESM2_ESM.pdf]

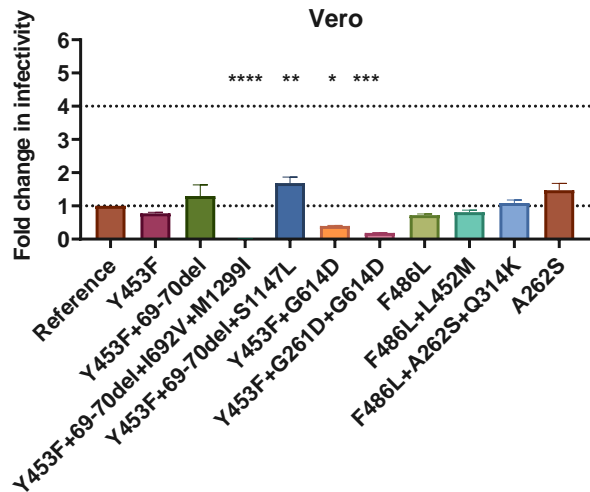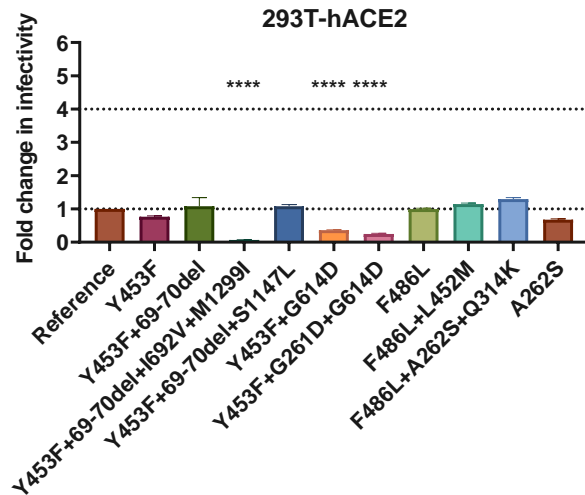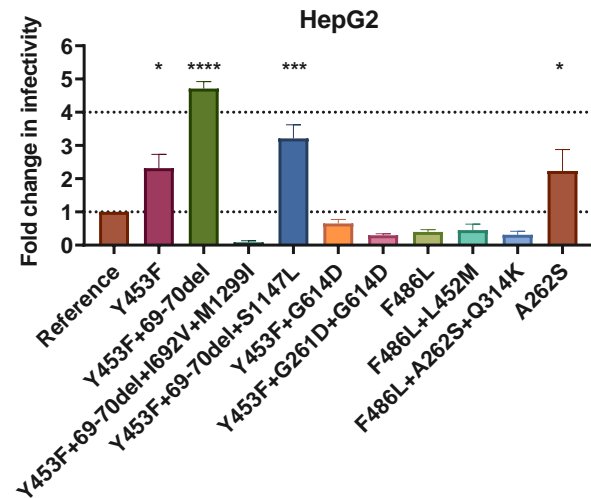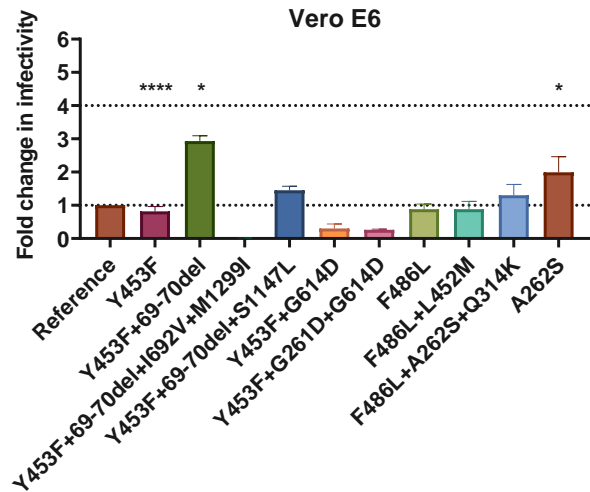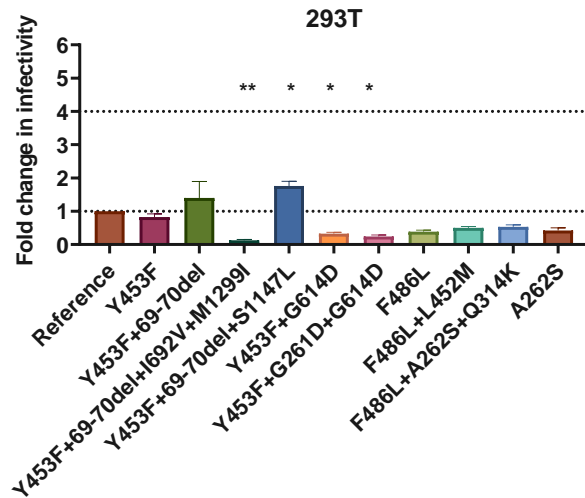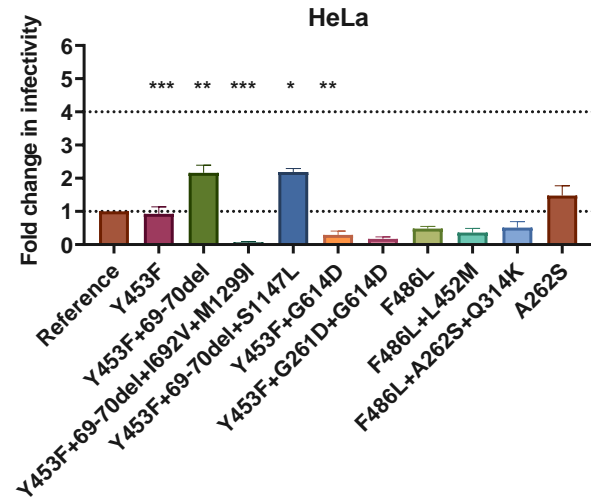

Supplement: Supplementary file 3 — Infection of 6 cell lines with mink variants [file 41392_2021_617_MOESM3_ESM.pdf]

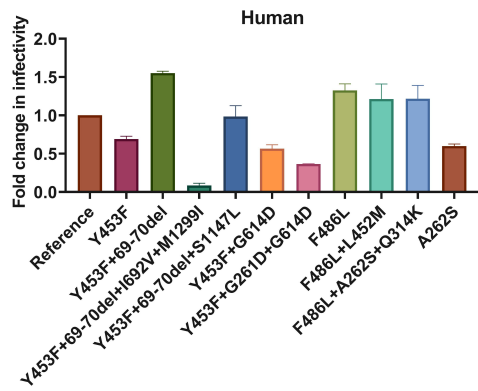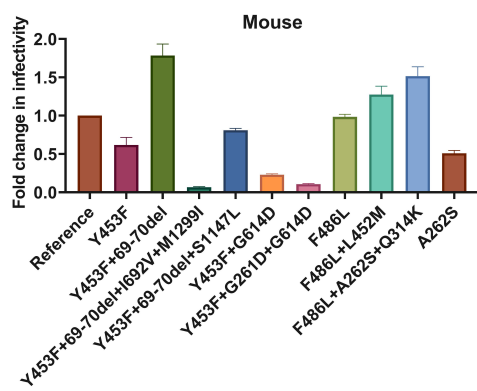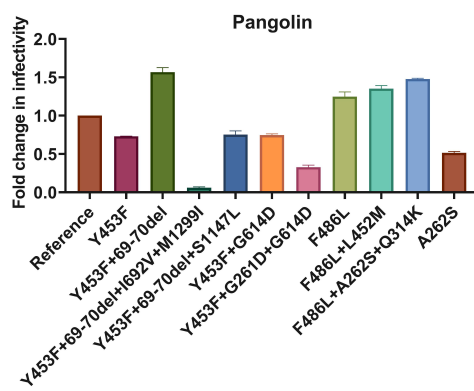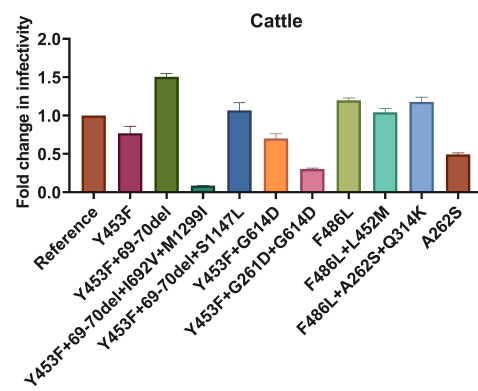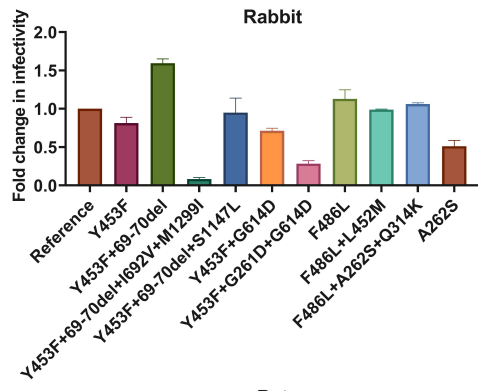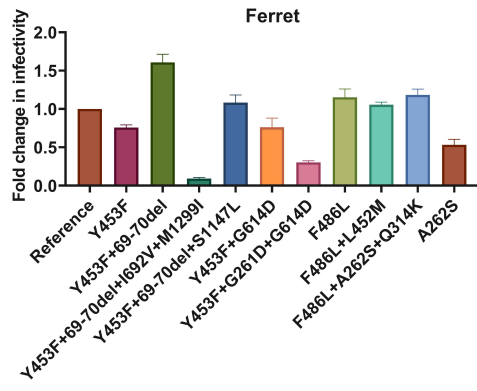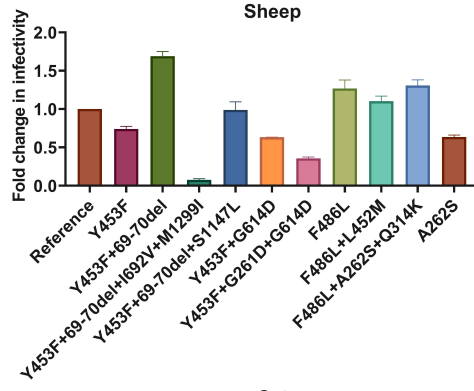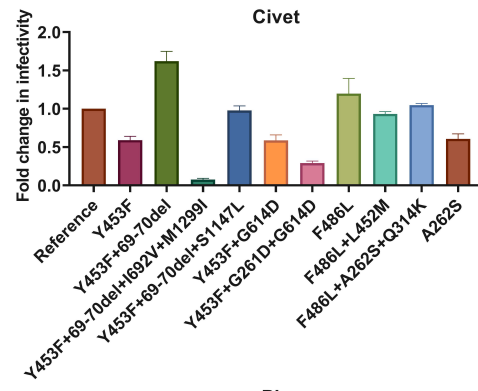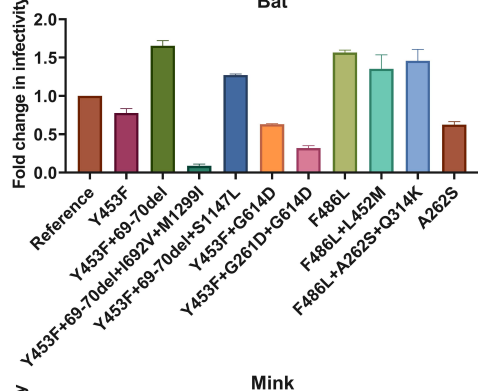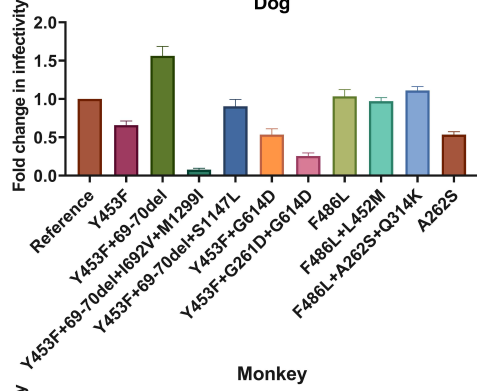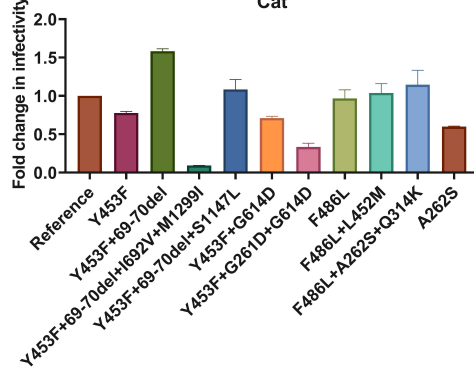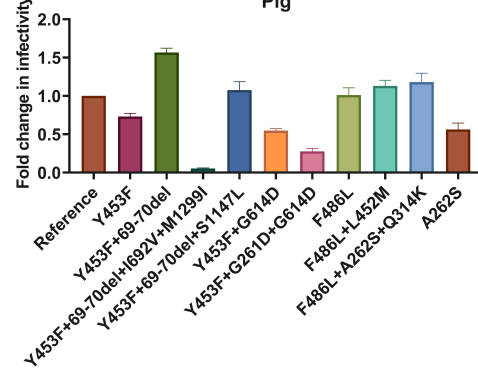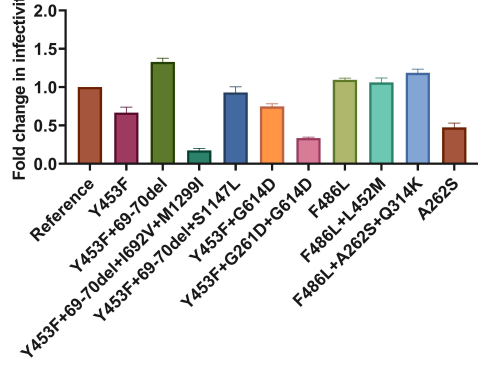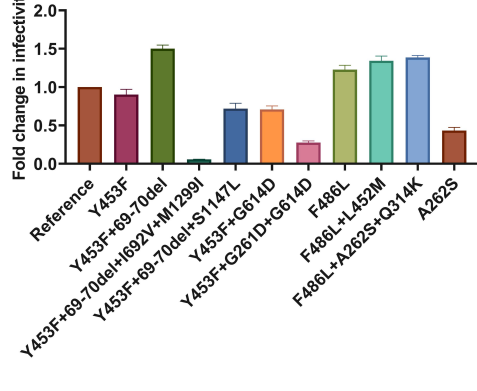

Supplement: Supplementary file 4 — Infection of 293T cells expressing with ACE2 from different species [file 41392_2021_617_MOESM4_ESM.pdf]

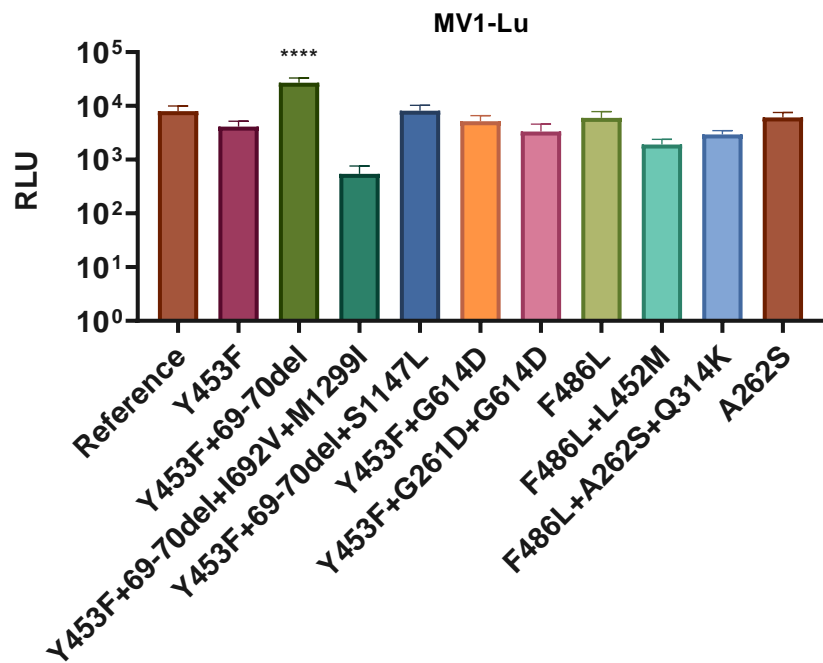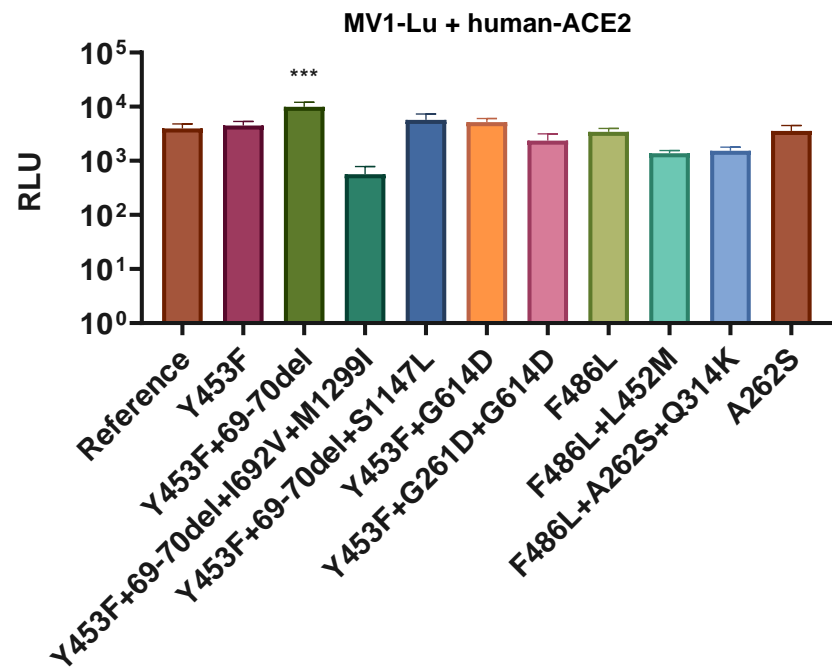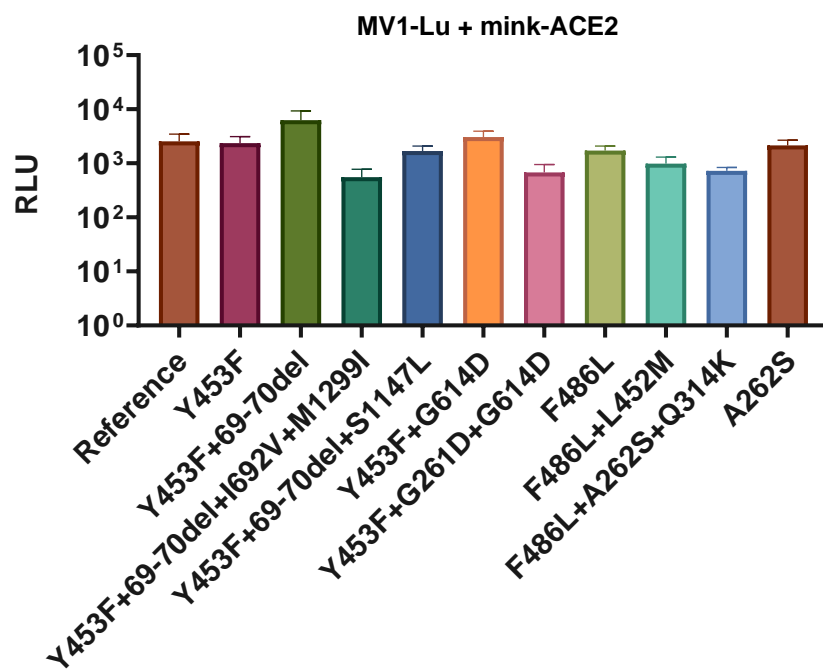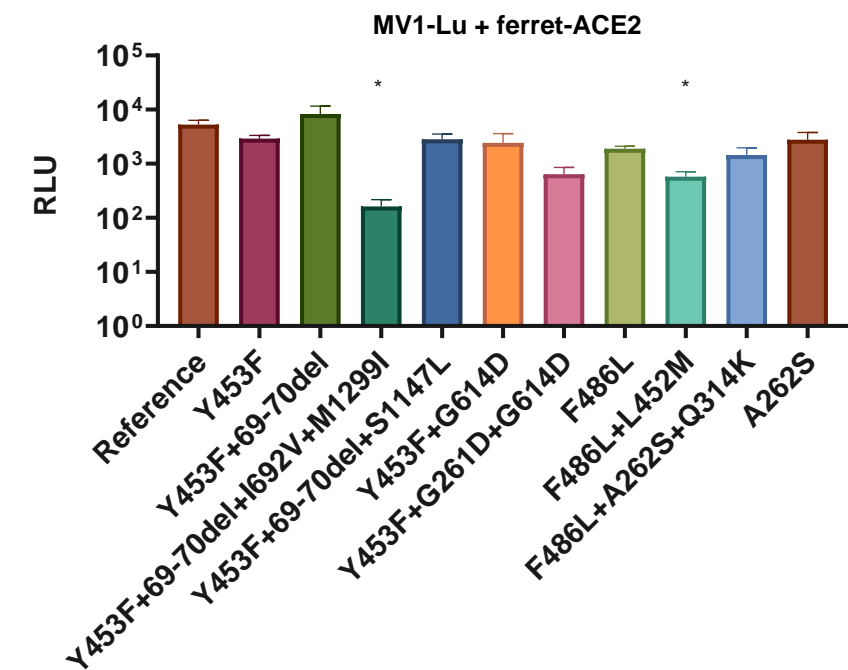

Supplement: Supplementary file 5 — Infection of MV1-lu cells expressing with human, mink or ferret ACE2 [file 41392_2021_617_MOESM5_ESM.pdf]

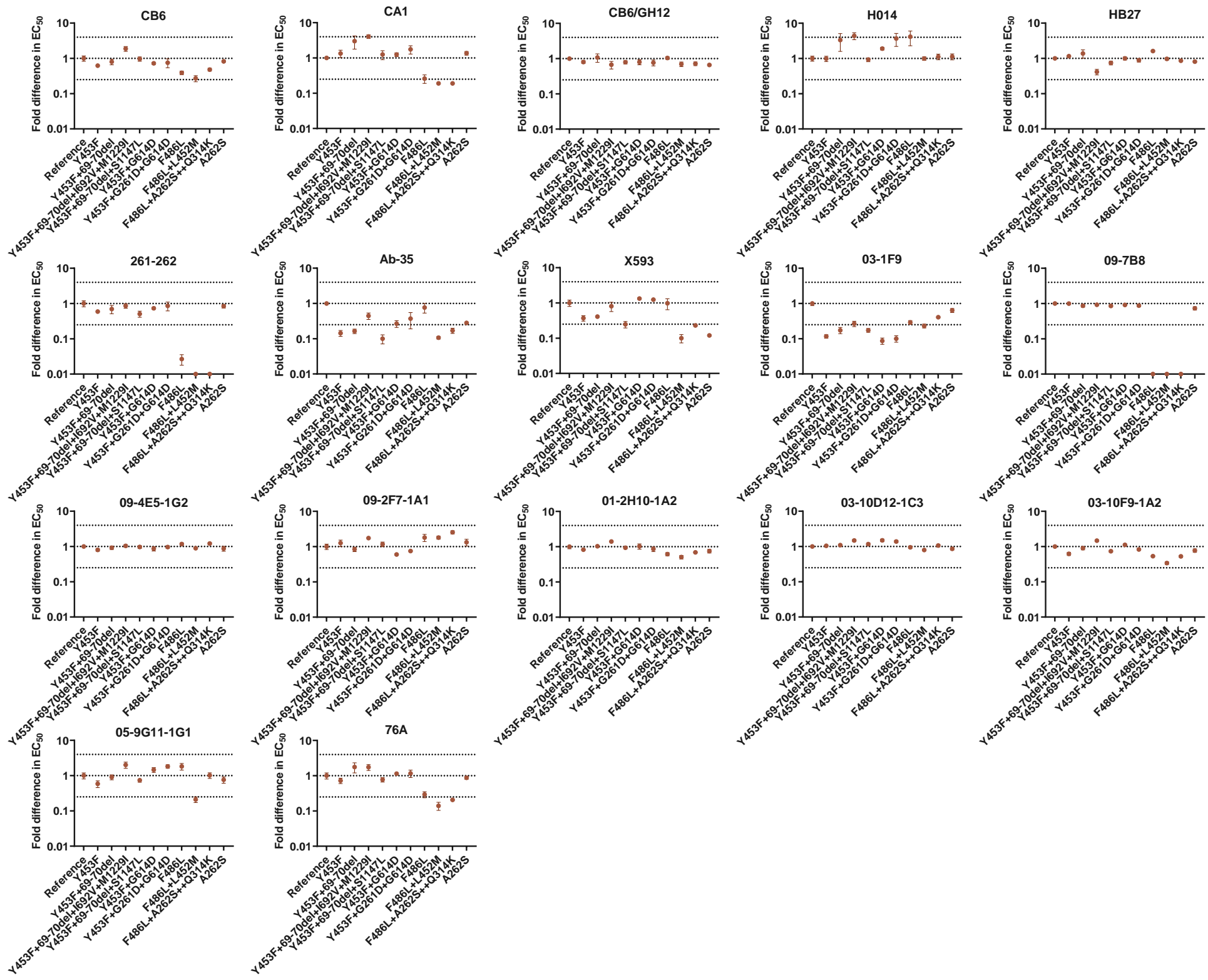

Supplement: Supplementary file 6 — The antigenicity analyses of mink variants using monoclonal antibodies [file 41392_2021_617_MOESM6_ESM.pdf]

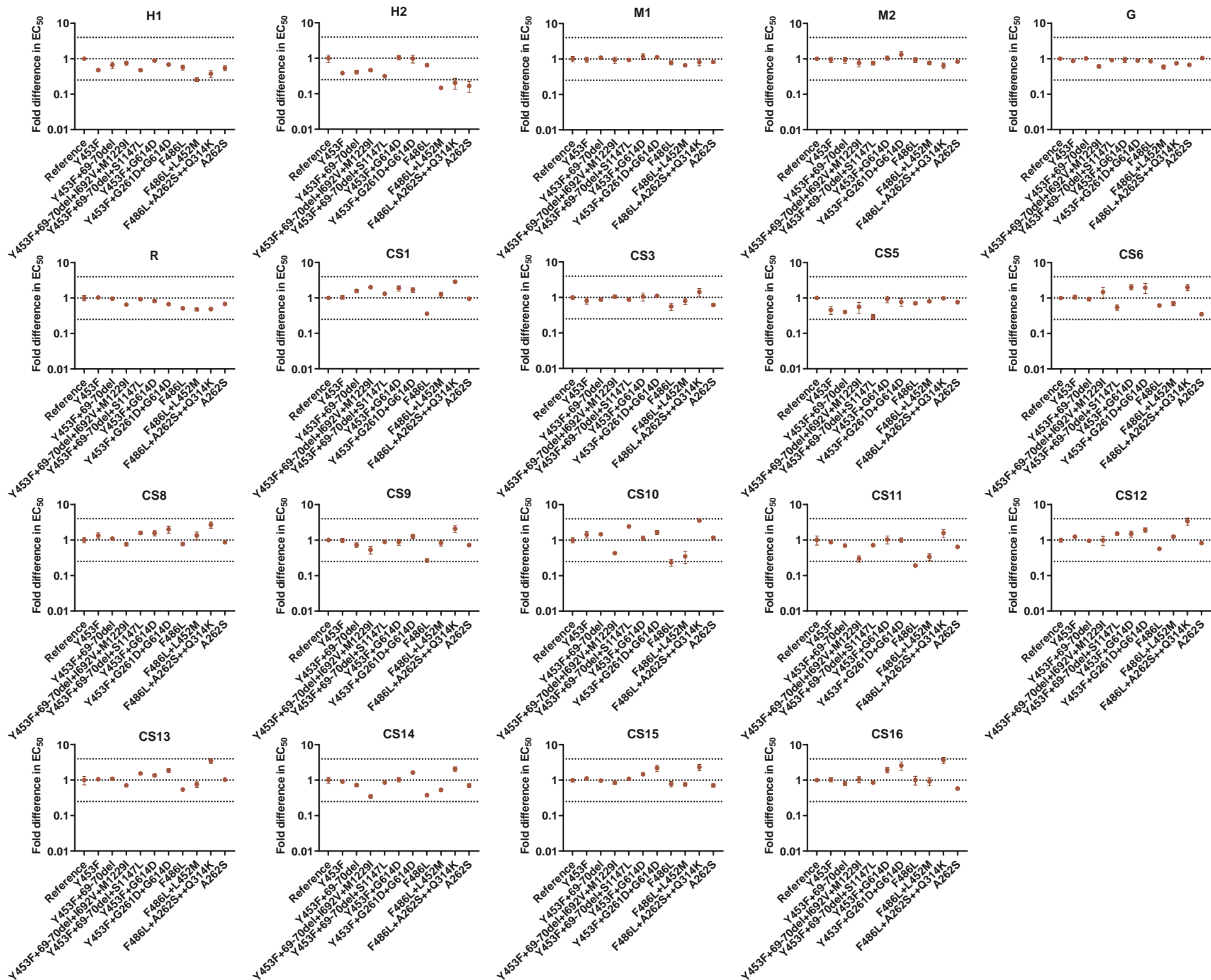

Supplement: Supplementary file 7 — The antigenicity of mink related SARS-CoV-2 using polyclonal antibodies and convalescence plasma [file 41392_2021_617_MOESM7_ESM.pdf]
